# Transurethral Radiofrequency Collagen Denaturation for Treatment of Female Stress Urinary Incontinence: A Review of the Literature and Clinical Recommendations

**DOI:** 10.1155/2012/384234

**Published:** 2011-10-12

**Authors:** James Chivian Lukban

**Affiliations:** Division of Urogynecology, Department of Obstetrics and Gynecology, Eastern Virginia Medical School, Norfolk, VA 23507, USA

## Abstract

Stress urinary incontinence is a prevalent condition in women with a significant negative effect on quality of life. Intervention includes behavioral modification, intravaginal devices, pelvic floor muscle exercises, biofeedback, functional electrical stimulation, and surgical procedures. We will review a new in-office procedure for the treatment of SUI that may serve as a viable nonsurgical option.

## 1. Introduction

Stress urinary incontinence (SUI) is estimated to affect up to 35% of adult women worldwide [1] and 15.7% of community-dwelling women domestically [2]. According to the National Hospital Discharge Survey, there are approximately 100,000 inpatient procedures performed for SUI in the United States annually, with outpatient procedures estimated to exceed 105,000 as per the 2006 National Survey of Ambulatory Surgery [3]. 

The International Continence Society defines SUI as “the complaint of involuntary leakage of urine upon effort or exertion, or on sneezing or coughing” [4]. In a European sample of 1573 women with urinary incontinence, >80% considered their symptoms to be bothersome, with a negative impact on quality of life as per the Incontinence Quality of Life Questionnaire (I-QOL) [5]. Risk factors for SUI include, but are not limited to, age, parity, history of hysterectomy, forceps delivery, and obesity.

Non-surgical treatment options include behavioral modification, intravaginal devices, pelvic floor muscle exercises, biofeedback, and functional electrical stimulation. In an assessment of 13 systematic reviews on conservative treatment of SUI, Latthe et al. concluded that pelvic floor muscle training (PFMT) was better than no treatment [6]. Nine-to-twelve-month success rates following PFMT with or without biofeedback and/or health education programs range from 52.6% to 74.8% [7–9]. Long-term durability in these patients is likely a function of life-long exercise. 

As there is no Food and Drug Administration (FDA) approved medical treatment for SUI, there is a resultant void in terms of invasiveness between conservative therapy and surgical intervention. Urethral bulking agents have shown benefit, however, are indicated for the treatment of SUI due to intrinsic sphincter deficiency (ISD), and are not universally administered in an office setting [10]. Renessa offers an office-based treatment for SUI in the absence of ISD through radiofrequency energy (RF) delivered transurethrally under local anesthesia. 

## 2. Pathogenesis of SUI

The pathogenesis of SUI is thought to be the result of urethral hypermobility secondary to a weakening or disruption of the pelvic floor musculature and/or pubourethral ligament, with a subsequent loss of pressure transmission from the bladder to the urethra upon provocation [11, 12]. As most women, independent of continence status, exhibit a measure of hypermobility, Blaivas et al. contend that SUI is dependent upon the concomitant presence of vesical neck funneling [13]. Ultrasound confirmation of funneling in 111 patients, as reported by Huang and Yang, was associated with a lower maximal urethral closure pressure (MUCP), a smaller area under the urethral pressure profile curve, a lower Valsalva leak point pressure (VLPP), and a larger volume of leakage on pad test [14].

## 3. Transurethral Radiofrequency Mechanism of Action

Transurethral RF treatment of the bladder neck and proximal urethra is thought to reduce funneling through the denaturation of submucosal collagen, with a resultant reduction in tissue compliance. Similar technology has proven effective in the treatment of patients with gastroesophageal reflux disease and fecal incontinence [15, 16]. In a preclinical porcine study employing transurethral RF, Valsalva leak point pressure was higher in the treatment group receiving 24 foci of RF energy at 65°C as compared to controls at 8 weeks (*P* = 0.06) [17].

## 4. Safety of Transurethral Radiofrequency

**Figure 1:**
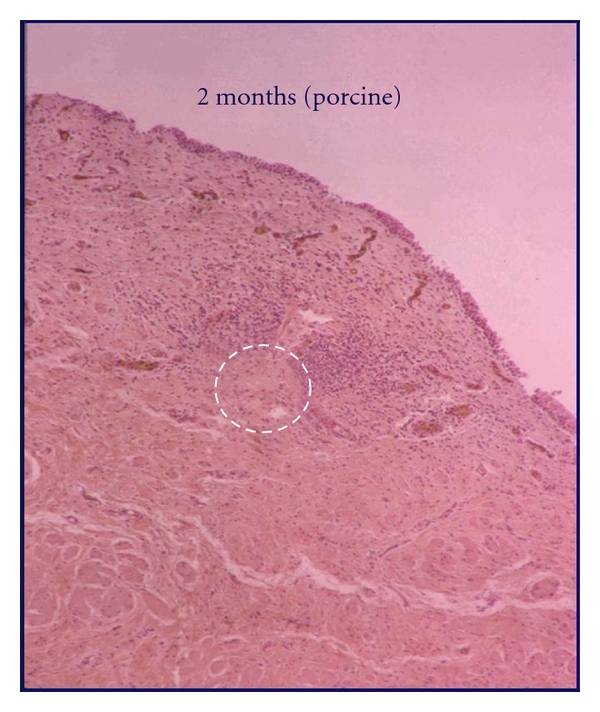
Porcine bladder neck posttreatment with Renessa. histologic image (hematoxylin and eosin) of porcine bladder outlet at 8 weeks following radiofrequency collagen remodeling. Denatured collagen is surrounded by focal chronic inflammatory cells.

Transurethral RF collagen denaturation differs from RF ablation in that subnecrotic temperatures are employed, resulting in collagen remodeling as opposed to necrosis [17]. Additionally, treated foci are microscopic, avoiding gross tissue destruction. In the aforementioned animal study by Edelstein, none of the 30 treated animals demonstrated obstruction or stricture within the proximal urethra or bladder neck. Histopathology after sacrifice revealed, most commonly, focal chronic inflammation within the submucosa 1-2 mm beneath the epithelium, with evidence of fibroplasia and vascular proliferation [17] (Figure 1). Created are localized regions of denatured collagen at each focus, approximately 200 *μ* in diameter [23].

## 5. The Renessa Device

**Figure 2:**
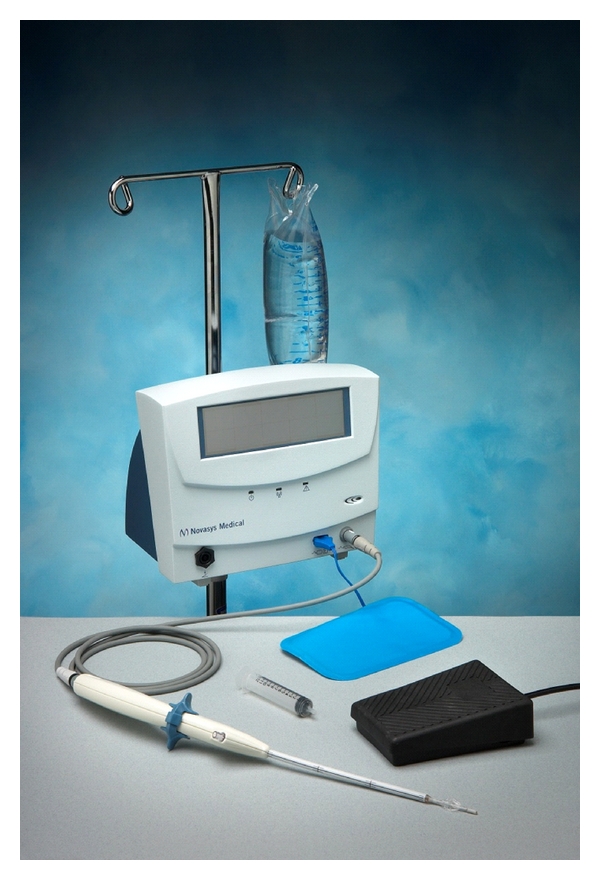
Renessa equipment.

**Figure 3:**
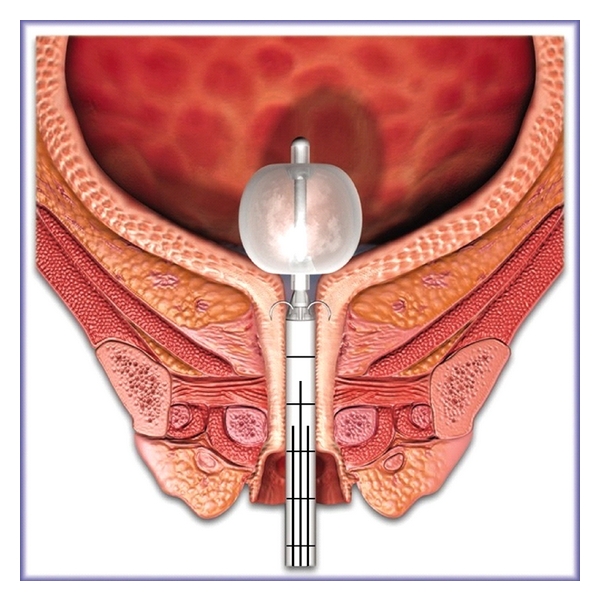
Probe with electrodes deployed delivering treatment.

Renessa is an FDA-approved device which includes an RF generator, a sterile single-use 21 F transurethral probe, foot pedal, probe interface cable, and standard AC power cord (Figure 2). Low-power RF energy is delivered through four partially insulated 23-gauge nickel-titanium needle electrodes deployed from the probe shaft into the submucosa of the bladder neck and proximal urethra (Figure 3). Tissue temperatures are measured automatically. Impedance is also reported prior to energy delivery to ensure appropriate contact between electrodes and tissue. Irrigation of the mucosa with sterile water occurs transurethrally throughout the duration of the procedure to prevent overheating of the mucosa and submucosa. Energy is delivered to a total of thirty-six sites circumferentially. 

## 6. Procedure Description

**Figure 4:**
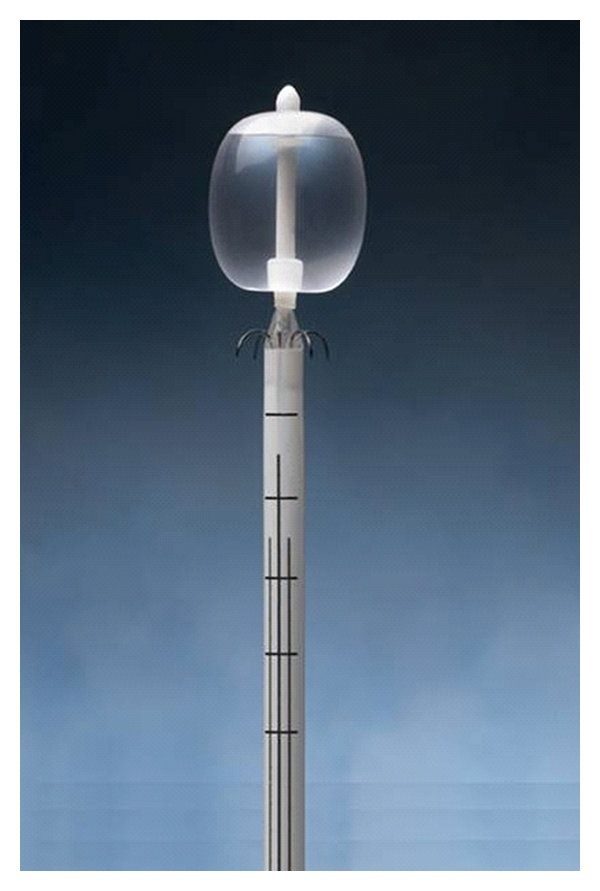
Probe with markings.

The radiofrequency probe is inserted until the tip is within the bladder lumen. The balloon is then insufflated with 10 cc of water. To treat the bladder neck, the electrodes are deployed first, and gentle traction along the previously determined urethral axis is applied (approximately 1/2 pound of force with the operator's index and middle fingers). The screen will display impedance upon initial pedal pressure. If all electrodes read less than 300 ohms, the pedal is pressed again to begin radiofrequency delivery. During treatment, the four electrodes traverse the mucosa and rest within the submucosa. Energy is delivered for a 60-second cycle while sterile room temperature water simultaneously irrigates the mucosa to prevent thermal injury. The submucosa immediately surrounding the four tips is heated and maintained at 65 degrees Celsius for a minimum of 30 seconds. The electrodes are withdrawn, and the probe shaft is repositioned after the first treatment cycle, first 30 degrees to the right, and then 30 degrees to the left of midline for cycles 2 and 3, respectively. Markings to guide such rotation are in the form of longitudinal lines on the probe shaft (Figure 4). The bladder neck receives a total of 12 discrete foci of denaturation.

To treat the proximal urethra, the same steps are carried out; however, traction is placed on the probe prior to deployment of the electrodes. Three cycles of energy are delivered to the proximal urethra followed by another 3 cycles just distal to the initial site, achieved with a slightly greater degree of traction. Thus, the proximal urethra receives a total of 24 foci of denaturation. 

## 7. Clinical Recommendations for Office-Based Lower Urinary Tract Anesthesia

The safe and effective administration of topical and local anesthesia must be fully considered, as it is essential for the successful completion of Renessa in the office. Wells and Lenihan reported on the feasibility of in-office anesthesia in patients undergoing transurethral radiofrequency treatment, employing preprocedure diazepam with a bilateral periurethral block using a total of 10 cc of 2% lidocaine [24]. Thirty-three women completed a visual analog scale (0 = no pain, 10  = terrible pain) immediately prior to discharge. Overall, 42% of patients rated their pain as 0, with a mean pain score of 1.4 ± 1.8. The following is a summary of the anesthetic regimen we currently employ.

### 7.1. Preprocedure Oral Regimen

The patient is instructed to take an anxiolytic such as diazepam 5–10 mg and a nonsteroidal such as ibuprofen 800 mg about 30–60 minutes before the procedure. 

### 7.2. Periurethral and Bladder Neck Topical Anesthesia

The introitus is prepared with povidone iodine. A 6-inch catheter is placed, and the bladder is drained. A negative urine dip is confirmed. Five cc of 2% xylocaine jelly is then infused into the catheter as it is withdrawn. EMLA cream is placed on a cotton swab and inserted transurethrally to rest at the bladder neck. The resting urethral angle with swab in place is determined by a goniometer to direct the orientation of the Renessa probe during treatment. 

A small aliquot of EMLA cream is also applied adjacent to the urethral meatus at 3 and 9 o'clock in preparation for injection of local anesthesia at these sites. Experience with the safety and efficacy of EMLA on the labia has been previously demonstrated [25].

### 7.3. Periurethral and Bladder Neck Injection Anesthesia

After 10 minutes, a periurethral block is performed with a total of 10 cc of 1% xylocaine using a 22 gauge, 1 1/4” needle introduced at the previously anesthetized 3 and 9 o'clock sites. The needle is buried to the hub (to ensure anesthesia of the bladder neck) along the urethral axis as determined by the cotton swab present within the urethra. It should be noted that the proximal urethra and bladder neck are well vascularized, circumscribed by a pampiniform plexus of veins. It is therefore essential that prior to injecting xylocaine, one aspirates the syringe to reduce the possibility of intravascular injection. Anesthetic is infused at each site in two aliquots −3 cc at the bladder neck followed by 1/2 cm withdrawal, aspiration and reinjection of the remaining 2 cc's. If additional anesthesia is required during treatment, another 5 cc of 1% xylocaine may be administered as above on either side. 

### 7.4. Intravesical Bladder Neck Anesthesia

Immediately after periurethral and bladder neck injection, intravesical anesthesia of the bladder neck is carried out. The cotton swab within the urethra is removed, and a red rubber catheter is placed to drain any residual urine. The bladder is retrograde filled with 30 cc of 1% xylocaine. The patient then stands or sits upright to ensure contact between the intravesical xylocaine and bladder neck. After 10 minutes, the bladder is drained completely, filled with 30 cc of sterile water at room temperature (to cool tissue during treatment), and the catheter removed. Complete evacuation of intravesical anesthesia is suggested, as xylocaine absorption may be significantly increased in highly vascularized traumatized areas such as the bladder neck and proximal urethra following treatment with Renessa.

### 7.5. Postprocedure Oral Regimen

Patients are given a prescription for phenazopyridine 200 mg three times a day for three days to provide lower urinary tract analgesia. The administration of postprocedure antibiotics may be at the discretion of the physician.

## 8. Clinical Data

A summary of prospective trials employing Renessa is presented in Table 1 [18–22]. Inclusion criteria common to all studies were SUI and urethral hypermobility, with exclusion of those with a history of previous anti-incontinence surgery and those with primary urge-associated leakage in the presence of mixed incontinence. In the Appell and Elser trials, patients with a LPP of <60 cm H_2_O on urodynamics were not eligible for participation [19, 21]. 

Regarding 12 to 18 month efficacy, patients treated with low-energy RF to 36 submucosal foci exhibited a ≥10 point score reduction in I-QOL ranging from 44% to 50.3%, and a ≥50% reduction in IEF ranging from 46.7% to 67%. An I-QOL score improvement of ≥10 points has been shown to correlate with patient perception of improvement as being “much better,” a ≥25% reduction in IEF, and a ≥25% reduction in stress pad weight [26]. Although no statistically significant difference was observed by Lenihan et al. between treatment and sham groups regarding a ≥10 point improvement in I-QOL, a subanalysis of patients deemed to have moderate-to-severe SUI determined that 74% of those receiving treatment versus 50% of those receiving sham achieved such improvement (*P* = 0.03) [27]. Additionally, outcomes in this population were independent of menopausal status.

Patients treated with transurethral RF collagen denaturation experienced rare long-term sequelae. No serious AE's were reported in any of the aforementioned clinical trials, and no difference was seen in the incidence of AE's between treatment and sham groups.

## 9. Discussion

Renessa represents an office intervention for SUI that is safe and is without significant AE's or known long-term negative effects. Safety has been confirmed in animal studies with no evidence of posttreatment urethral obstruction or stricture formation. The putative mechanism of decreased funneling may be supported by both animal and human data in which LPP was found to improve following RF treatment [17, 19]. 

Efficacy of transurethral collagen denaturation appears to be within range of that of PFMT. Alewijnse et al. reported 1-year success following PFMT (with randomization of 129 patients to PFMT alone versus PFMT plus one of three health education programs), citing a ≥50% improvement in IEF in 74.8% (64.4% by intent to treat) of patients overall [7]. 

In terms of actual number of leaks, Elser et al. reported a reduction in median weekly IEF from a 15.0 (1.0 − 245.0) to 7.5 (0.0 − 140.0) at 12 months (*P* = 0.0026) [21]. This is in line with data from the Alewijnse trial in which was reported a reduction in mean weekly IEF from 22.9 ± 24.1 to 7.8 ± 12.2 at 12 months (*P* < 0.001). In a large RCT of 530 patients randomized to individual or group PFMT, Janssen et al. reported similar data at 9 months, with a reduction in mean weekly IEF from 16.3 ± 15.8 to 8.6 ± 15.5 and from 14.4 ± 15.3 to 6.1 ± 10.5 for the individual and group patients, respectively, [8].

Eighteen-month data following Renessa shows improvements at 12 months to be durable [22]. This may be an advantage over PFMT, as durability following pelvic floor muscle rehabilitation may be wholly dependent upon continued therapy. 

We chose not to compare our data to bulking agents, as Renessa is indicated for SUI and not ISD. Additionally, as transurethral RF treatment is in-office, we did not compare such therapy to surgical anti-incontinence data.

## 10. Conclusion

Radiofrequency collagen denaturation is a safe, nonsurgical, and in-office procedure for the treatment of female SUI, providing an improvement in quality of life. Such therapy may represent an alternative to PFMT or for those who have failed such treatment. 

## 11. Coding

The current procedural terminology code (CPT) for the Renessa procedure is 53860.

## Figures and Tables

**Table 1:** Summary of Renessa clinical studies.

Study (year)	Design	Patients (number)	Mean age (range)	Follow-up (months)	Measures	Outcomes	Adverse events (% incidence)
Sotomayor and Bernal (2005) [18]	Pilot clinical trial with sequential enrollment into 1 of 4 groups based on number of submucosal foci. Group 1–24 Group 2–36 Group 3–48 Group 4–60	41	47.6 (34–81)	12	I-QOL IEF	I-QOL—incidence of >10 point score improvement. Group 1: 63% Group 2: 44% Group 3: 70% Group 4: 67% Mean scores showed significant improvement for Groups 2–4. IEF—incidence of ≥50% reduction. Group 1: 63% Group 2: 67% Group 3: 70% Group 4: 89%	Urgency (22) Dysuria (8)

Appell et al. (2006) [19]	Randomized sham-controlled trial.	173 (110 Treated) (63 Sham)	50 (22–76)	12	I-QOL LPP (cm H_2_O)	I-QOL—incidence of ≥10 point score improvement. Treated: 48% Sham: 44% (*P* = 0.7) LPP—mean ± SD Treated: 13.2 ± 39.2 Sham: −2.0 ± 33.8 (*P* = 0.02)	AE (Rx, Sham incidence) Wet OAB (10, 9.5) Dysuria (9.1, 1.6) Dry OAB (7.3, 3.2) UTI (4.5, 4.8) Asymptomatic DO (1.8, 6.3) Retention (0.9, 0) Hematuria (0.9, 0) Hesitancy (0, 1.6)

Appell et al. (2007) [20]	Retrospective followup of 12-month RCT	21 (Treated)	52.2 (39.0–65.4)	36	I-QOL IEF	I-QOL—mean improvement: 12.7 points IEF—incidence of ≥50% reduction: 56%	No new AE's

Elser et al. (2009) [21]	Prospective single-arm study	136 (ITT)	47.0 (26.0–87.0)	12	I-QOL IEF UDI-6 PGI-I PWT	I-QOL—incidence of ≥10 point score improvement: 50.3% Mean scores showed significant improvement (*P* < 0.0001) IEF—incidence of ≥50% reduction: 50% UDI-6—mean scores showed significant improvement (*P* < 0.0001) PGI-I—improvement: 49.6% (“very much” 14.4%, “much” 14.4%, “a little” 20.8%) PWT—≥50% reduction: 69% (45% < 1 gram)	Dysuria (5.2) Retention (4.4) Pain (2.9) UTI (2.9) Increased leakage (0.7)

Elser et al. (2010) [22]	Prospective single-arm study	136 (ITT)	47.0 (26.0–87.0)	18	I-QOL IEF UDI-6 PGI-I	I-QOL—incidence of ≥10 point score improvement: 47.8% Mean scores showed significant improvement (*P* < 0.0001) IEF—incidence of ≥50% reduction: 46.7% UDI-6—mean scores showed significant improvement (*P* < 0.0001) PGI-I—improvement: 50.4% (“very much” 9.6%, “much” 15.2%, “a little” 25.6%)	No new AE's

I-QOL: Incontinence quality of life instrument, IEF: incontinence episode frequency, LPP: leak point pressure, SD: standard deviation, Rx: treated group, AE: adverse event, OAB: overactive bladder, UTI: urinary tract infection, DO: detrusor overactivity, RCT: randomized controlled trial, UDI-6: urogenital distress inventory, PGI-I: patient global impression of improvement, PWT: pad weight test, and ITT: intent to treat.
